# A modified assay for the enumeration of ascaris eggs in fresh raw sewage

**DOI:** 10.1016/j.mex.2017.04.001

**Published:** 2017-05-01

**Authors:** Esmaeil Shahsavari, Jonathan Schmidt, Arturo Aburto-Medina, Basma Khallaf, Vivek Balakrishnan, Nicholas D. Crosbie, Aravind Surapaneni, Andrew S. Ball

**Affiliations:** a Centre for Environmental Sustainability and Remediation, School of Science, RMIT University, Bundoora, Victoria 3083, Australia; b ALS Water, 22 Dalmore Drive, Scoresby, Victoria 3179, Australia; c Melbourne Water, 990 La Trobe Street, Docklands, Victoria 3008, Australia; d South East Water, 101 Wells Street, Frankston, Victoria 3199, Australia

**Keywords:** Helminths, *Ascaris suum*, Recovery rate, Wastewater

## Abstract

Soil-transmitted helminths (STHs) pose a significant public health problem, infecting approximately 2 billion people globally. Despite relatively low prevalence in developed countries, the removal of STHs from wastewater remains crucial to allow the safe use of biosolids or recycled water for agriculture. Wastewater helminth egg count data can contribute to an assessment of the need for, or success of, a parasite management program. Although the World Health Organisation (WHO) has recommended a standard method for counting helminth eggs in raw sewage based on the method of Bailenger (Ayres et al., 1996), the method generally results in low percentage egg recoveries. Given the importance of determining the presence of STHs, it is essential to develop novel techniques that optimise the recovery rate of eggs from raw sewage. In the present study:

• The method described by Bowman et al. (2003) was optimized for the concentration and enumeration of helminth eggs in raw sewage from municipal sewage treatment plants.
• The method is simple and reproducible and recovers a greater percentage of helminth eggs compared to the WHO method.

## Graphical abstract

**Figure:**
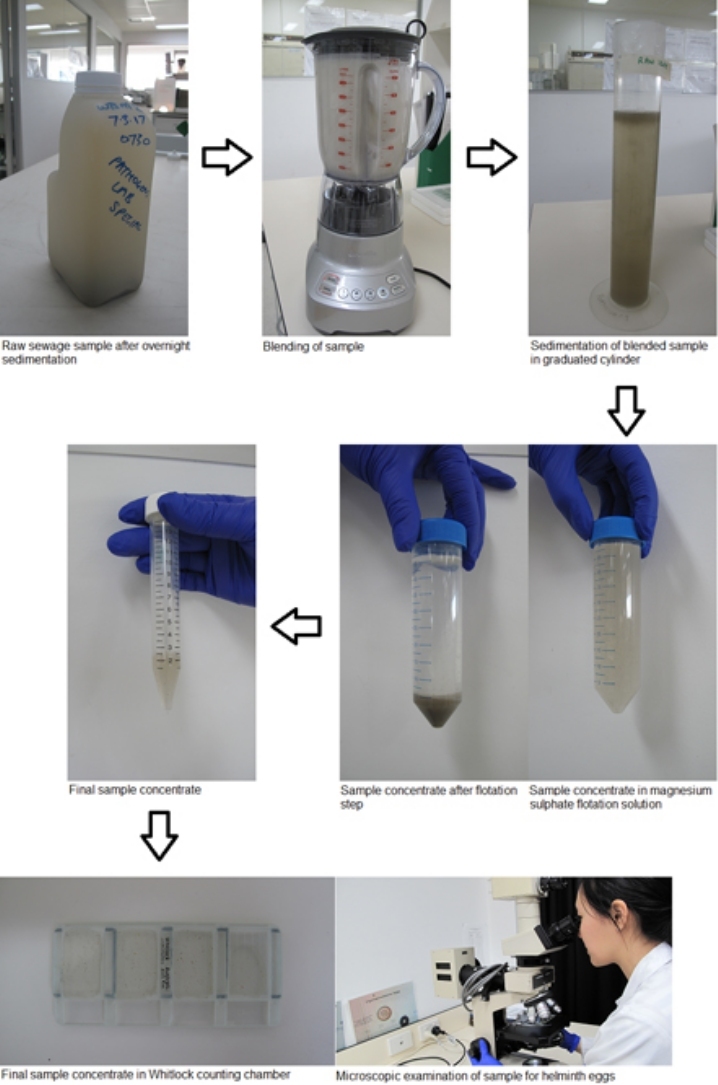

## Method details

### The new method (Modified Bowman Method)

**Fig. 1:**
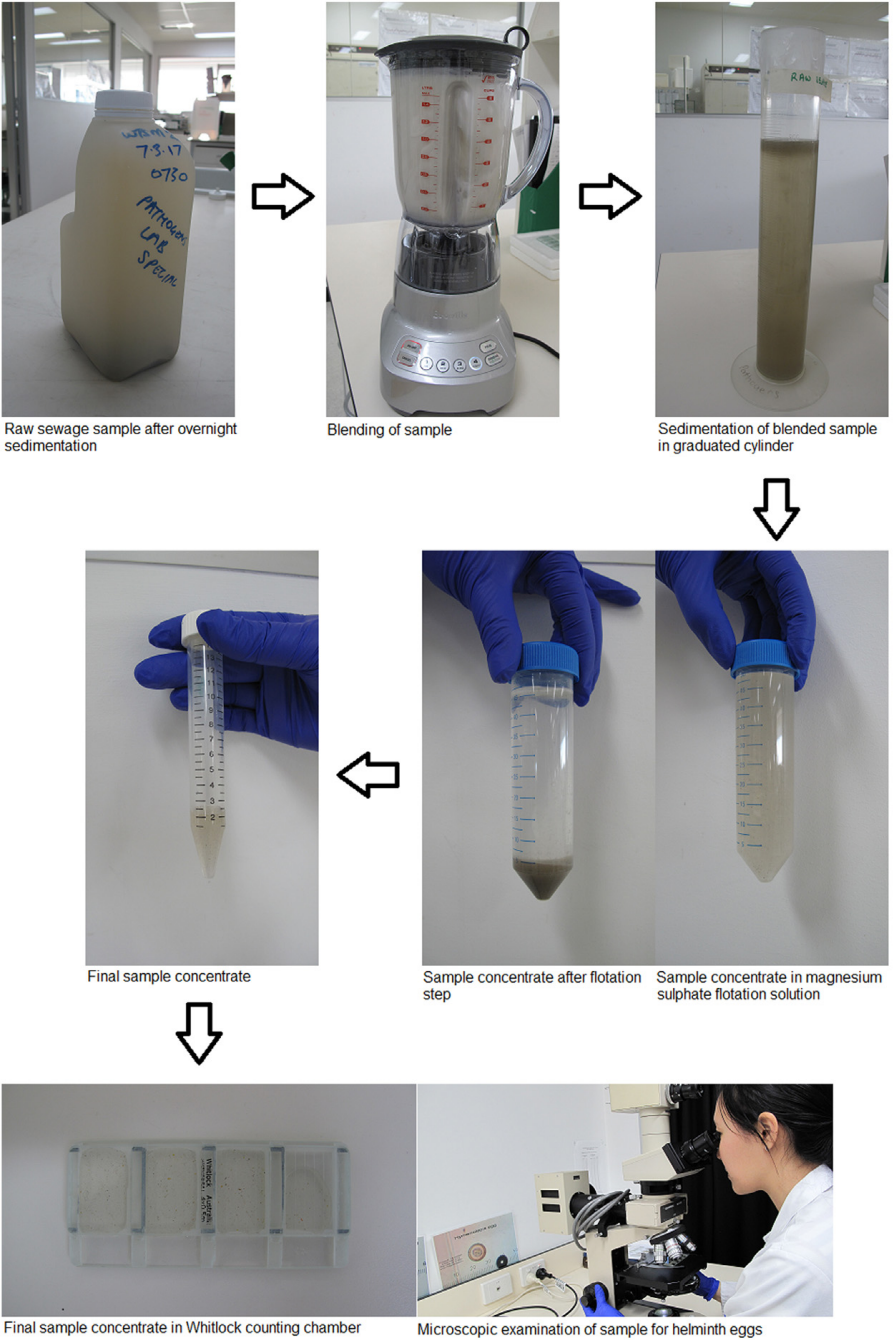
The outline of the protocol (modified Bowman method).

*Note*: Original Bowman method and WHO protocol details can be found in Refs. [1] and [2] (http://www.who.int/water_sanitation_health/wastewater/labmanual.pdf) respectively.

The main outlines of the protocol are presented in Fig. 1.

1. Allow the raw sewage (1 L) sample to sediment in the sample bottle overnight at 4 °C.
2. Aspirate and discard the supernatant to just above the sediment layer (∼100 mL volume remaining) and resuspend the sediment by vortexing and transfer to a blender.
3. Rinse the sample bottle twice with ∼100 mL of 1% 7× detergent each rinse (MP Biomedicals) and transfer and combine each rinse to the blender in step 2. Add sterile reagent water to a final volume of 300 mL if necessary.
4. Add 1 mL antifoam-B (Sigma-Aldrich) to the sample and blend on high speed for 1 min.
5. Transfer the homogenized sample into a tall 1000 mL graduated cylinder. Rinse the blender twice with ∼100 mL of 1% 7× detergent each rinse and transfer each rinse into the graduated cylinder. Add additional 1% 7× detergent to a final volume of ∼900 mL.
6. Allow sample to sediment overnight or a minimum of 4 h at 4 °C.
7. Aspirate and discard the supernatant to just above the sediment layer (∼100 mL volume) and resuspend the sediment by vortexing and transfer to a blender.
8. Rinse the graduated cylinder twice with ∼100 mL of 1% 7× detergent each rinse and transfer each rinse to the blender in step 7. Add sterile reagent water to a final volume of 300 mL if necessary.
9. Add 1 mL antifoam-B to the sample and blend on high speed for 1 min.
10. Repeat steps 6 through 9 allowing the sample to sediment in the blender (three blending cycles in total).
11. Allow sample to sediment in the blender overnight or a minimum of 4 h at 4 °C.
12. Aspirate and discard the supernatant to just above the sediment layer (∼100 mL volume) and transfer the sediment to a 250 mL conical centrifuge tube.
13. Rinse the blender with ∼50 mL of 1% 7× detergent each rinse and transfer each rinse to the centrifuge tube in step 12. Add 1% 7× detergent to a final volume of 250 mL if necessary.
14. Centrifuge the sample in a swinging bucket rotor at 800 × *g* for 10 min at ambient temperature.
15. Aspirate and discard the supernatant to just above the sample pellet.
16. Transfer the sample pellet to a 50 mL centrifuge tube. If the pellet is greater than 5 mL then evenly distribute the pellet into multiple 50 mL centrifuge tubes containing no more than 5 mL of pellet in each tube.
17. Add 50 mL MgSO_4_ (specific gravity 1.25) and resuspend the sample pellet by vortexing.
18. Centrifuge the sample in a swinging bucket rotor at 800 × *g* for 10 min at ambient temperature.
19. Transfer the supernatant containing the helminth eggs to a second 250 mL conical centrifuge tube.
20. Repeat steps 16 and 17.
21. Transfer and combine the supernatant containing the helminth eggs to the centrifuge tube in step 18.
22. Add sterile reagent water to a final volume of 250 mL and centrifuge the sample in a swinging bucket rotor at 800 × *g* for 10 min at ambient temperature.
23. Aspirate and discard the supernatant.
24. Resuspend the pellet in at least five volumes of MgSO_4_ (specific gravity 1.25).
25. Mix the final concentrate by vortexing and transfer ∼0.5 mL aliquots into each chamber of a Whitlock Universal 4 chamber worm egg counting slide.
26. Leave the prepared slide to stand on a flat surface for 5 min before examination to allow the eggs to float to the surface.
27. Examine the entire sample at 50× or greater magnification.

### Method validation

*Ascaris suum* eggs were prepared from infected pig faeces by Excelsior Scientific (USA). An aliquot of the eggs was diluted in phosphate buffered saline to a final mean concentration of 100 eggs per 100 μL (based on triplicate counts). Raw sewage samples were collected from different sewage treatment plants in Victoria, Australia and 1 L homogenous samples were prepared for processing. Samples were processed in parallel, 1) following the modified Bowman method with the addition of 100 μL of the stock *A. suum* egg preparation, 2) following the modified Bowman method without the addition of 100 μL of the stock *A. suum* egg preparation, and 3) following the WHO method [2] with the addition of 100 μL of the stock *A. suum* egg preparation. The percentage recovery of the spiked *A. suum* eggs following the modified Bowman and the WHO methods were calculated as the number of eggs counted in the corresponding spiked sample less the number of indigenous eggs counted in the sample that was not spiked. No indigenous *Ascaris* spp. eggs were detected for those samples processed without the addition of the spiked *A. suum* eggs.

The percentage recoveries of *A. suum* eggs following the modified Bowman method are presented in Table 1. The results showed that the recoveries varied between 14 and 87% with a mean recovery of 57%. For the comparison of the WHO and modified Bowman methods the mean recoveries were 11 and 42%, respectively (Table 2). The greater mean percentage recovery of helminth eggs following the modified Bowman method compared to the WHO method was statistically significant (*p*-value < 0.0001, paired *T*-test), which demonstrates that the modified Bowman method gives more accurate results in evaluating the concentration of helminth eggs from raw sewage.

**Table 1:** Percentage recoveries of spiked *A. suum* eggs from different raw sewage samples using the modified Bowman method.

Source of raw sewage samples	Recovery rate (%)
Boneo Treatment Plant, Boneo	87
Pakenham Treatment Plant, Pakenham	50
Mt Martha Treatment Plant, Mt Martha	84
Somers Treatment Plant, Somers	66
Eastern Treatment Plant, Carrum	18
Eastern Treatment Plant, Carrum	61
Eastern Treatment Plant, Carrum	14
Western Treatment Plant, Werribee	64
Western Treatment Plant, Werribee	40
Western Treatment Plant, Werribee	52
Western Treatment Plant, Werribee	83
Western Treatment Plant, Werribee	69
Western Treatment Plant, Werribee	74
Western Treatment Plant, Werribee	38
Mean	57
Range	14–87

**Table 2:** Comparison of percentage recoveries of spiked *A. suum* eggs from different raw sewage samples processed following the WHO and modified Bowman methods.

Source of raw sewage samples	Recovery rate (%)	
	WHO method	Modified Bowman method
Boneo Treatment Plant, Boneo	11	51
Pakenham Treatment Plant, Pakenham	10	43
Mt Martha Treatment Plant, Mt Martha	16	53
Somers Treatment Plant, Somers	12	49
Longwarry Treatment Plant, Longwarry	9	29
Koo Wee Rup Treatment Plant, Koo Wee Rup	11	38
Blind Bight Treatment Plant, Blind Bight	11	30
Mean	11	42

The method described by Bowman et al. [1] was optimized for the concentration and enumeration of helminth eggs in raw sewage with a significantly improved egg recovery rate compared to the WHO method [2]. The modified Bowman method is a simple, reproducible and low cost method for routine use by commercial laboratories and researchers.
